# Kidney Disease and Hematopoietic Stem Cell Transplantation

**DOI:** 10.34067/KID.0000000692

**Published:** 2025-01-09

**Authors:** Amanda DeMauro Renaghan, José Maximino Costa, Alexandra Esteves

**Affiliations:** 1 Division of Nephrology, University of Virginia Health System, Charlottesville, Virginia; 2 Nephrology Department, Instituto Português de Oncologia do Porto, Porto, Portugal; 3 Nephrology Department, Centro Hospitalar Universitário de Coimbra, Coimbra, Portugal

**Keywords:** AKI, cancer, chronic kidney failure, stem cell, transplantation

## Abstract

Hematopoietic stem cell transplantation (HSCT) is a potentially curative therapy for patients with hematologic malignancies and certain solid tumors and nonmalignant hematologic conditions. Both AKI and CKD occur commonly after HSCT and are associated with significant morbidity and mortality. AKI and CKD in this setting may result from direct effects of the transplant or be caused by pretransplant bone marrow conditioning regimens and/or nephrotoxic agents administered in the post-transplant period. In this article, we review the epidemiology, risk factors, etiologies, pathophysiology, diagnosis, prevention, and treatment of post-HSCT AKI and CKD, with special attention to recent advances in this fast-moving and evolving field.

## Introduction

Hematopoietic stem cell transplantation (HSCT) involves conditioning of recipient bone marrow (BM) followed by engraftment of stem cells harvested from BM, peripheral blood (PB), or umbilical cord blood (UCB). Transplanted cells may come from the affected patient (autologous) or a related/unrelated donor (allogeneic). Patients receiving myeloablative autologous or allogeneic HSCT receive intensive conditioning regimens (high-dose chemotherapy±total body irradiation) to eradicate the underlying cancer and create niches for cell engraftment. Patients not appropriate for myeloablative conditioning because of age/comorbidities may be considered for nonmyeloablative (NMA) or reduced intensity conditioning (RIC).^1^ Importantly, allogeneic (but not autologous) HSCT requires post-transplant immunosuppression to prevent graft-versus-host disease (GVHD); calcineurin inhibitors (CNIs, *i.e*., tacrolimus, cyclosporine) represent the cornerstone of GVHD prophylaxis at many centers.^2,3^ Removal of donor T-cell or specific T-cell subpopulations from the graft through physical separation or antibody-based techniques (T-cell depletion [TCD]) offers opportunities to reduce the risk of GVHD and reduce or eliminate the need for intensive GVHD prophylaxis.^4^

## AKI after HSCT

In this article, we cover general epidemiology, causes, and management of AKI after HSCT. We subsequently dive deeper into the pathophysiology, evaluation, and management of several key etiologies of post-HSCT AKI, including GVHD, capillary leak and engraftment syndromes (ESs), hepatic sinusoidal obstruction syndrome/veno-occlusive disease (SOS/VOD), transplant-associated thrombotic microangiopathy (TA-TMA), and viral infections.

### Epidemiology

AKI is common after HSCT, with one recent meta-analysis reporting 55% pooled incidence of any AKI and 8% incidence of severe AKI.^5^ Risk varies by patient characteristics, kidney reserve, conditioning/donor type, and AKI definition.^3^ Myeloablative allogeneic HSCT carries greater risk of AKI (19%–66%) compared with NMA allogeneic HSCT (29%–54%), likely reflecting the greater immunosuppression and higher incidence of SOS/VOD after myeloablative conditioning.^6^ AKI incidence is lowest after autologous HSCT (AHSCT; 10%–29%), potentially related to more rapid engraftment and absence of GVHD/CNI exposure.^7,8^ UCB recipients are at especially high AKI risk, in part because of delayed engraftment and subsequent infection.^9–13^

Incidence of AKI requiring KRT (AKI-KRT) varies widely by patient characteristics, transplant type (allogeneic>autologous), and timeframe relative to cell infusion. In one recent study of 616 allogeneic HSCT recipients, AKI-KRT occurred in 3% of patients within 100 days of transplant, with 43% recovering kidney function.^14^ Risk of AKI-KRT is greatest among critically ill patients, with a 2023 cohort study of adult allogeneic recipients admitted to the intensive care unit within 1 year of transplant reporting an AKI-KRT incidence of 21%; 87% died within 90 days of AKI-KRT onset.^15^

Newer data confirm the associations between post-transplant AKI and increased short-term and long-term mortality.^5,8,9,14–21^ AKI onset/severity represent key risk factors for post-transplant CKD, which itself is associated with early mortality.^9,14,20–22^

A summary of AKI risk factors identified in recent studies is provided in Table 1.

**Table 1:** Risk factors for post-transplant AKI identified in recent studies

Allogeneic	Autologous
• Higher HCT-CI • Hypertension • Hypoalbuminemia • CNI use • Supratherapeutic tacrolimus levels • TA-TMA • SOS/VOD • BK cystitis • Recurrent CMV reactivations • Bacterial infection • Grade 3–4 aGVHD	• Higher HCT-CI • CKD, lower baseline eGFR • Obesity • Amyloidosis • Higher involved free light chain • Higher urinary NAG • Mucositis grade 3–4 • Exposure to nephrotoxic drugs • Diuretic use

Refs. 14,16–19. aGVHD, acute graft-versus-host disease; CMV, cytomegalovirus; CNI, calcineurin inhibitor; HCT-CI, hematopoietic cell transplantation-specific comorbidity index; NAG, *N*-acetyl-*β*-d-glucosaminidase; SOS/VOD, sinusoidal obstruction syndrome/veno-occlusive disease; TA-TMA, transplant-associated thrombotic microangiopathy.

### Etiologies

**Figure 1:**
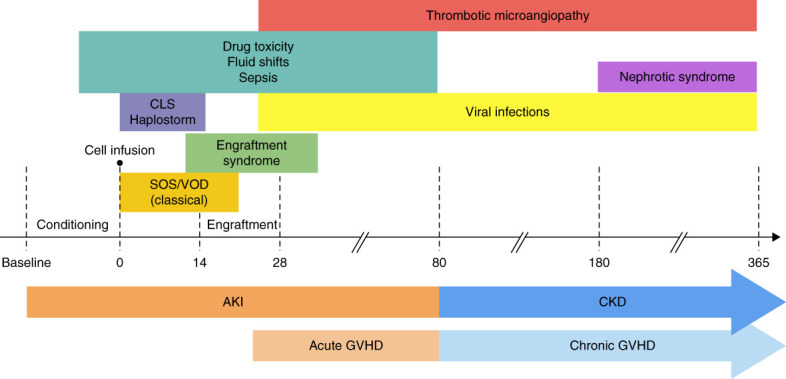
**Kidney injury after hematopoietic-cell transplantation.** From N Engl J Med, Sangeeta Hingorani, M.D., M.P.H., Renal Complications of Hematopoietic. *Cell Transplantation*, *374*, 2256–2267. Copyright © (2016) Massachusetts Medical Society. Reprinted with permission from Massachusetts Medical Society.^140^ CLS, capillary leak syndrome; GVHD, graft-versus-host disease; SOS/VOD, sinusoidal obstruction syndrome/veno-occlusive disease.

Patients undergoing HSCT are exposed to multiple nephrotoxic insults, and AKI is often multifactorial. Risk and potential etiologies of AKI vary by time from cell infusion (Figure 1). Myeloablative and RIC regimens have been associated with AKI, both indirectly (mucositis/vomiting/diarrhea causing prerenal AKI) and through direct nephrotoxicity.^23–28^ HSCT recipients are prone to sepsis, which may induce AKI through hemodynamic changes and endothelial injury, compounded by the need for nephrotoxic antibiotics/antivirals.^29^ CNI-induced AKI is usually hemodynamic, resulting from afferent arteriolar vasoconstriction and subsequent ischemic tubular injury.^30^ CNIs may also cause TA-TMA.^31^

### Evaluation/Diagnosis

Evaluation of post-transplant AKI includes thorough history and physical, urinalysis/microscopy, and quantification of proteinuria (spot urine albumin-to-creatinine [Cr] ratio, urine protein-to-Cr ratio).^2,3^ Workup for transplant-specific causes should be considered in clinical context (see below). Biopsy may be useful for unexplained AKI or proteinuria.^2^

Patients undergoing HSCT experience changes in muscle mass that influence Cr-based GFR estimates.^32,33^ Accordingly, there is interest in identifying biomarkers to detect subclinical kidney damage and facilitate early AKI diagnosis. In 2022, Malyszko and colleagues measured levels of four urinary biomarkers of kidney injury (*IGFBP7*, *TIMP2*, netrin-1, semaphorin A2) ≥3 months after HSCT. All biomarkers (in subgroups with eGFRs above and below 60 ml/min per 1.73 m^2^) were significantly higher in HSCT recipients relative to healthy volunteers, suggesting kidney injury despite normal/near-normal kidney function by Cr-based eGFR.^34^ While a recent statement from the American Society of Onco-Nephrology advocates for the use of CKD-EPICr-Cys in patients with cancer, these experts acknowledge the need for additional studies in patients with hematologic malignancies given the paucity of data regarding the performance of cystatin C in this population.^35^

### Prevention/Treatment

Pretransplant, choice of conditioning regimen and donor source should be tailored to individual disease status/comorbidities.^17,36^ RIC may be considered in high-risk patients.^17^ A recent study by Abramson *et al.* demonstrated lower AKI incidence after T-cell–depleted HSCT as compared with unmodified transplant (tacrolimus-, tacrolimus-/sirolimus-, or post-transplant cyclophosphamide-based).^14^ Madsen and colleagues showed reduced incidence of AKI among patients receiving dual TCD with antithymocyte globulin and post-transplant cyclophosphamide for GVHD prophylaxis, possibly related to lower rates of acute GVHD (aGVHD) in the TCD group.^17^ Together, these data suggest that TCD may be an appropriate preventive strategy in selected patients.

Peritransplant, strict management of fluid balance is critical. Nephrotoxic medications and iodinated contrast should be used judiciously, CNI dosing/levels adjusted closely, and infections and GVHD treated promptly.^17,37–39^

Treatment of post-HSCT AKI is largely supportive. Care must be taken to prevent/mitigate fluid overload.^3,40^ No consensus guidelines exist regarding best timing of dialysis initiation after HSCT.

### GVHD

GVHD occurs when alloreactive donor T cells cause immune-mediated organ injury.^41–43^ GVHD has been associated with prerenal AKI (mucositis/vomiting/diarrhea in gut GVHD) and identified as a risk factor for TA-TMA. While the kidney has not traditionally been recognized as a primary GVHD target, data from preclinical/clinical studies support the existence of kidney GVHD through direct tubulointerstitial injury.^36,44^

### Endothelial Injury Syndromes

**Figure 2:**
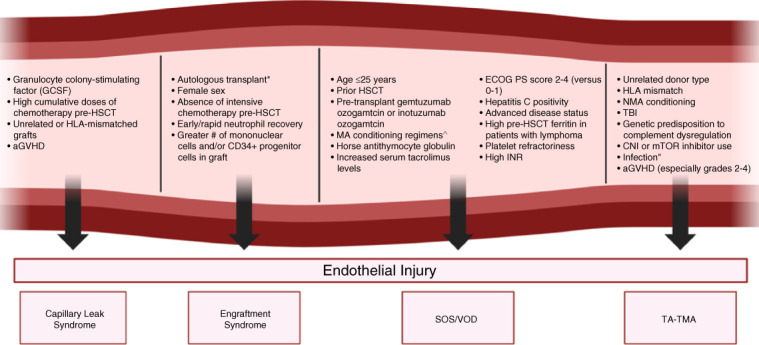
**Risk factors for EISs.** *Particularly for breast cancer, lymphomas other than Hodgkin lymphoma, POEMs, multiple sclerosis. ^TBI-based and busulfan-based. **Systemic, BK virus, CMV, parvovirus B19, adenovirus. Refs. 52,54,64,83,91,141–143. aGVHD, acute graft-versus-host disease; CMV, cytomegalovirus; CNI, calcineurin inhibitor; ECOG, Eastern Cooperative Oncology Group; EIS, endothelial injury syndrome; GCSF, granulocyte colony-stimulating factor; HSCT, hematopoietic stem cell transplantation; INR, international normalized ratio; mTOR, mammalian target of rapamycin; MA, myeloablative; NMA, nonmyeloablative; POEMs, polyneuropathy, organomegaly, endocrinopathy, monoclonal plasma cell disorder, skin changes syndrome; PS, performance status; TA-TMA, transplant-associated thrombotic microangiopathy; TBI, total body irradiation.

Endothelial activation/dysfunction after HSCT may be triggered by patient-specific and transplant-specific factors. Endothelial damage caused by proinflammatory, prothrombotic, and proapoptotic pathways may manifest as a group of clinical syndromes referred to as endothelial injury syndrome (Figure 2).^45^ In addition to chemoradiation-induced and drug-induced endothelial injury, alloreactive T lymphocytes likely play major roles in pathogenesis, leading to overlap between the development of endothelial injury syndrome and presence of GVHD and complicating diagnosis/management.^46,47^

## Cytokine Release and Capillary Leak Syndromes

Capillary leak syndrome (CLS) is an early post-HSCT complication and a severe manifestation of the larger cytokine release syndrome (CRS), driven by cytokine-mediated capillary endothelial injury and characterized by increased capillary permeability.^48,49^ Incidence varies widely by patient-specific and transplant-specific factors, but is generally considered to occur in approximately 5% of patients undergoing HSCT.^50^ Patients present within 15 days of transplant with weight gain, diuretic-resistant ascites, and pleural/pericardial effusions, with hemodynamic collapse and multiorgan dysfunction syndrome (MODS) in severe cases.^45,48^ Kidney injury occurs because of intravascular volume depletion and possibly direct inflammatory effects.^51,52^

ES is a form of CLS that occurs around time of neutrophil recovery, most commonly after AHSCT.^53,54^ AKI has been reported in up to 93% of patients with ES, with 52% developing stage 3 AKI and 28% requiring KRT.^53^ Pathophysiology is mediated by massive release of proinflammatory cytokines, products of neutrophil degranulation and oxidative metabolism, and systemic endothelial damage.^48^ Owing to their anti-inflammatory properties which may attenuate endothelial damage, corticosteroids represent the mainstay of therapy for CLS/ES.^45,48,51,52,55,56^

*Haplostorm* is a unique form of CRS that occurs within 14 days of transplantation in patients undergoing T-cell replete haploidentical HSCT.^12^ High incidence of CRS in this context has been attributed to the inherent mismatch of haploidentical transplants. Recipients of haploidentical PB transplants, as compared with BM transplants, seem to be most vulnerable because of the significantly increased number of lymphocytes in PB allografts.^57^ Research is ongoing regarding the role of the IL-6 inhibitor tocilizumab in this context.^58^

### Hepatic SOS/VOD

SOS/VOD is reported in approximately 14% of HSCT recipients and is most common after allogeneic transplant.^59^ Mortality in severe SOS/VOD with MODS approaches 85%. Pathophysiology is driven by injury to sinusoidal endothelial cells causing venular thrombosis/fibrosis and portal hypertension and by coagulative necrosis of hepatocytes. In the recently published CECinVOD multicenter study, circulating endothelial cells were significantly elevated in patients who developed SOS/VOD after allogeneic HSCT, reinforcing the role of endothelial injury and highlighting the potential role of circulating endothelial cell level as a diagnostic biomarker.^60^

Patients with SOS/VOD classically present within 21 days of transplant; late-onset cases also occur.^61^ Presentation is characterized by edema, hepatomegaly, and diuretic-resistant ascites. AKI is believed to be hemodynamic; acute tubular injury may occur with prolonged ischemia.^62^ Diagnostic criteria were updated by the European Society for Blood and Marrow Transplantation in 2023, aiming to enhance early detection (Table 2).^63^

**Table 2:** Revised European Society for Blood and Marrow Transplantation criteria for diagnosis of SOS/VOD (2023)

Probable	Clinical	Proven
Two of the following criteria must be present	Bilirubin ≥2 mg/dl and two of the following criteria must be present	Histologically proven SOS/VOD or hemodynamically proven (HVPG ≥10 mm Hg)
• Bilirubin ≥2 mg/dl	• Painful hepatomegaly	
• Painful hepatomegaly	• Weight gain >5%	
• Weight gain >5%	• Ascites	
• Ascites		
• Ultrasound and/or elastography suggestive of SOS/VOD		
**Onset**		
In the first 21 d after HSCT: classical SOS/VOD	>21 d after HSCT: late onset SOS/VOD	

For any patient, these symptoms/signs should not be attributable to others causes. HSCT, hematopoietic stem cell transplant; HVPG, hepatic venous pressure gradient; SOS/VOD, sinusoidal obstruction syndrome/veno-occlusive disease.

Ref. 63.

Recent studies have implicated a number of transplant-related and patient-related risk factors in the development of SOS/VOD (Figure 2).^64^ Despite the absence of a clear pathophysiologic mechanism, patients aged 25 or younger carry highest risk.^65^

Several agents have been investigated for prevention and treatment of SOS/VOD.^66–69^ Data regarding the value of ursodeoxycholic acid for prevention are inconclusive; however, prophylactic administration has been associated with less liver toxicity, less aGVHD, and improved survival, supporting use.^66,70^ The antithrombotic/fibrinolytic agent defibrotide is US Food and Drug Administration approved for the treatment of severe SOS/VOD. Based on pediatric clinical trial data and retrospective adult studies, some experts have recommended defibrotide for prevention of SOS/VOD in patients at very high risk; however, a recent randomized multicenter phase 3 trial (HARMONY) failed to show prophylactic benefit.^71,72^

In patients with suspected/established SOS/VOD, exposure to hepatotoxic/nephrotoxic drugs should be minimized.^3,73^ Clinicians should aim to maintain intravascular volume and kidney perfusion while avoiding overload.^73^ Limited newer data suggest benefit of transjugular hepatic portosystemic shunt in patients with very severe or progressive SOS/VOD refractory to medical therapy; additional studies are needed.^45,74^ When KRT is indicated in patients with SOS/VOD, continuous renal replacement therapy may be preferred.^75–78^

### Acute TA-TMA

TA-TMA is a multisystem disease associated with high morbidity/mortality. TA-TMA most commonly manifests as microangiopathic hemolytic anemia with thrombocytopenia and kidney dysfunction (CKD>AKI); however, patients may also develop bowel ischemia, diffuse alveolar hemorrhage, seizure, or cutaneous vasculitis. Reported incidence varies widely (8%–39%) because of heterogeneous criteria and underrecognition.^79,80^

**Figure 3:**
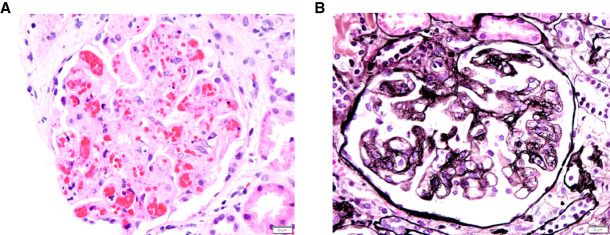
**Histology of TA-TMA.** (A) Glomerulus with features of acute TMA including endothelial swelling, red blood cell fragmentation, karyorrhexis, and associated mesangiolysis (hematoxylin and eosin stain, original magnification 400×, scale bar=20 *µ*m). (B) Glomerulus with features of chronic TMA including mesangiolysis and double contour formation (Jones methenamine silver stain, original magnification 400×, scale bar=20 *µ*m). TA-TMA, transplant-associated thrombotic microangiopathy; TMA, thrombotic microangiopathy.

Pathophysiology of TA-TMA is driven by the triad of endothelial cell activation, complement dysregulation, and microangiopathic hemolytic anemia.^81^ Patients with renal TA-TMA typically present 4–12 months after HSCT with hypertension and slowly rising serum Cr; however, some may demonstrate earlier or more fulminant presentations. Renal histology is reviewed in Figure 3. The Modified Jodele Criteria have gained support among international experts (Table 3). High-risk TA-TMA (hrTMA) is associated with increased nonrelapse mortality and defined by presence of any high-risk feature. Per International TA-TMA Working Group consensus, patients with hrTMA should be considered for early TA-TMA directed therapy.^82^

**Table 3:** Modified Jodele criteria and Transplant-Associated Thrombotic Microangiopathy Working Group definition of high-risk thrombotic microangiopathy

TA-TMA Criteria	
Biopsy-proven disease (kidney or gastrointestinal) or	
Clinical criteria: must meet ≥4 of the following seven criteria within 14 d at two consecutive time points	
Anemia^a^	Defined as one of the following
	1. Failure to achieve transfusion independence for pRBCs despite evidence of neutrophil engraftment
	2. Hemoglobin decline from patient's baseline by 1 g/dl
	3. New onset of transfusion dependence
Thrombocytopenia	Defined as one of the following
	1. Failure to achieve platelet engraftment
	2. Higher than expected platelet transfusion needs
	3. Refractoriness to platelet transfusions
	4. 50% reduction or greater in baseline platelet count after full platelet engraftment
Elevated LDH	>ULN for age
Schistocytes	Present
Hypertension	>99th percentile for age (<18 yr), or systolic BP ≥140 mm Hg or diastolic BP ≥90 mm Hg (≥18 yr)
Elevated sC5b-9	≥ULN
Proteinuria	≥1 mg/mg rUPCR

hrTMA Definition
Defined by *any one* of the following
• Elevated sC5b-9 (≥ULN)
• rUPCR ≥1 mg/mg
• Elevated LDH (≥2 times ULN)
• Concurrent grade 2–4 aGVHD
• Concurrent infection (bacterial or viral)
• Organ dysfunction

Ref. 82. aGVHD, acute graft-versus-host disease; hrTMA, high-risk transplant-associated thrombotic microangiopathy; LDH, lactate dehydrogenase; pRBCs, packed red blood cells; PRCA, pure red cell aplasia; rUPCR, random urine protein to creatinine ratio; sC5b-9, soluble C5b-9; TA-TMA, transplant-associated thrombotic microangiopathy; ULN, upper limit of normal.

a Rule out other causes of anemia, such as autoimmune hemolytic anemia and pure red cell aplasia.

Risk factors for TA-TMA are highlighted in Figure 2.^83^ aGVHD is a major risk factor, with emerging data suggesting that TA-TMA may represent an endothelial complication of GVHD.^31,84–90^ Genetic susceptibility to complement dysregulation may increase risk in specific patients.^91^

Management of suspected/confirmed TA-TMA includes BP control (often with renin-angiotensin-aldosterone-inhibiting agents), transfusion support, and dialysis as indicated.^83^ Precipitating factors (*i.e*., viral infections, GVHD) should be addressed accordingly. In patients receiving CNIs or mammalian target of rapamycin inhibitors, replacement with alternative GVHD prophylaxis may be appropriate.^92^ Plasma exchange may be beneficial in selected patients (*i.e*., presence of Factor H antibodies).^31,93^ Both defibrotide and the anti-CD20 mAb rituximab have shown potential benefit; large-scale prospective studies are lacking.^83,94–96^

Given the role of complement activation as a fundamental driver of TA-TMA, agents targeting the complement system have gained significant attention.^97^ Greatest experience comes from use of the C5 inhibitor eculizumab.^31,98–100^ Data from the first multi-institutional prospective study of eculizumab as an early targeted intervention in children/young adults with hrTMA/MODS demonstrated 71% survival 6 months after hrTMA diagnosis and 62% survival 1 year after transplant.^31,101^ Individualized dosing is critical, particularly in patients with significant bleeding.^102^ Studies investigating use of ravulizumab (C5 inhibitor with longer *t*_1/2_) and nomacopan (C5 and leukotriene B4 inhibitor) are ongoing.^97^

Because TA-TMA involves activation of all three complement pathways, lectin pathway inhibitors may be effective therapies.^97,103^ A recent single-arm open-label pivotal trial of 28 adult HSCT patients showed a 68% survival rate 100 days after TA-TMA diagnosis after narsoplimab administration.^103^ Given the high morbidity/mortality associated with TA-TMA, further research is urgently needed.

### Viral Infections

BK virus reactivation may cause hemorrhagic cystitis, urinary tract obstruction (clot formation, ureteral stenosis), or interstitial nephritis (BK virus-associated nephropathy).^104–108^ Risk is highest among haploidentical and UCB recipients after myeloablative conditioning.^104,107^ Symptomatic BK infection is associated with kidney function decline and worse survival.^109^ Adenovirus may cause acute tubulointerstitial disease; however, hemorrhagic cystitis is more common.^36,110^ Pediatric patients, those receiving T-cell depleted, HLA-mismatched, or UCB grafts, and patients with GVHD are at highest risk.^27^ Both BK and adenovirus have been associated with development of TA-TMA.^26^ Treatment options are limited by nephrotoxicity and/or lack of efficacy.^111,112^

While cytomegalovirus (CMV) nephritis is uncommon, other organ involvement (*i.e*., pneumonitis, colitis) carries significant morbidity, and CMV reactivation has been associated with increased mortality.^37^ CMV seropositive recipients from CMV seronegative donors, patients with GVHD, and those receiving grafts from unrelated or mismatched donor sources are at increased risk.^37,113^ Letermovir has shown safety and efficacy in preventing CMV reactivation, making this drug particularly attractive for prophylaxis in high-risk patients.^17,37,38^ Given toxicities associated with antiviral drugs (ganciclovir/valganciclovir-marrow suppression; foscarnet-kidney toxicity), risk of CMV disease must be balanced with risks of therapy.^38^

## CKD and HSCT

### Epidemiology

Patients with previous CKD present unique challenges in HSCT (Table 4).^114^ In our review, for uniformity, we defined CKD as GFR ≤60 ml/min per 1.73 m^2^ and/or proteinuria ≥300 mg/g. The effect of CKD on eligibility for HSCT, clinical course, and outcomes depends on the type of transplant. RIC is used mainly for older patients and those with pre-existing end-organ damage, including CKD.^6^ As immunosuppression is not required, AHSCT is better tolerated than allogeneic protocols. This is particularly true in multiple myeloma, where treatment (consolidation) with AHSCT is an established procedure for patients with CKD and ESKD.^115–118^

**Table 4:** Risk factors contributing to and challenges in treating CKD in patients undergoing hematopoietic stem cell transplant

Risk Factors Contributing to CKD
Patient-related factors • Older patients and other comorbidities • Female sex • Previous kidney dysfunction
Transplant-related factors • Type of conditioning regimen (mostly TBI) • AKI in the first 100 d post-HSCT (any cause) • Acute or chronic GVHD • CMV and BK virus infection, adenovirus reactivation • Use of CNIs • New-onset proteinuria • Acute or chronic TA-TMA

Challenges in Treating CKD
Management of volume
Choosing and adjusting drugs/dosing (especially in dialysis and CRRT patients)
Prophylaxis and treatment of infection and GVHD with nephrotoxic drugs Antibiotics • Vancomycin, aminoglycosides, ciprofloxacin Antifungals • Amphotericin B Antivirals • Foscarnet, acyclovir, cidofovir, ganciclovir, valacyclovir Immunosuppression CNIs, methotrexate
Logistics of KRT (hemodialysis and CRRT) in isolated patients

Refs. 6,121. CMV, cytomegalovirus; CNI, calcineurin inhibitor; CRRT, continuous renal replacement therapy; GVHD, graft-versus-host disease; HSCT, hematopoietic stem cell transplant; TA-TMA, transplant-associated thrombotic microangiopathy; TBI, total body irradiation.

Reported incidence of CKD after HSCT is highly variable (18%–66%), partially because of varying CKD definitions.^6,119,120^ Risk factors for post-transplant CKD are summarized in Table 4.^6,121^ Importantly, any post-transplant AKI, regardless of etiology, may lead to post-transplant CKD. Development of CKD after HSCT portends a worse prognosis, with increased risks of hospitalization and cardiovascular events, and about 4% progressing to ESKD.^6,122^

### Etiologies

There are four main causes that lead to the development of CKD post-HSCT: TA-TMA, CNI toxicity, nephrotic syndrome (NS), and chronic interstitial nephritis (CIN).

#### Chronic TA-TMA

**Figure 4:**
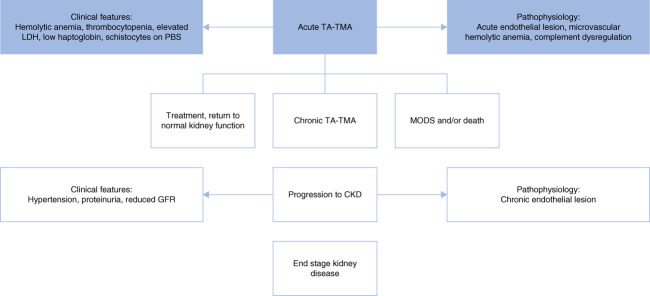
**Evolution of acute TA-TMA to chronic TA-TMA and progression to CKD**. LDH, lactate dehydrogenase; MODS, multiorgan dysfunction syndrome; PBS, peripheral blood smear.

Clinical manifestations of acute TA-TMA are reviewed above. Acute TA-TMA can evolve into a chronic form (as shown in Figure 4). Chronic microangiopathy may appear later (>100 days post-HSCT) with more insidious manifestations. New-onset hypertension is the first indicator. Appearance of proteinuria is an important marker of endothelial damage. Increasing Cr is subsequent, progressive, and even irreversible (Table 5).^81^ Importantly, hematologic manifestations of TA-TMA may not be present. Definitive diagnosis and degree of chronicity are best evaluated by kidney biopsy; however, risk of bleeding may preclude tissue diagnosis (Figure 3).

**Table 5:** Time of onset, clinical features/symptoms, and diagnosis of the main etiologies of posthematopoietic stem cell transplant CKD

Etiology	Onset (Post-HSCT)	Clinical Features/Symptoms	Diagnosis
Chronic TA-TMA	Uncertain (more than 100 d); after acute TA-TMA	• Hypertension • Proteinuria • Sustained kidney injury	Presumptive • History of prior acute TA-TMA • Anemia • High LDH • Low eGFR Definitive • Kidney biopsy
CNI nephropathy	After 3 mo	• Progressive kidney failure • Hypertension • Proteinuria	• Chronic CNI therapy • Exclusion of other causes (infections, glomerular disease) • Kidney biopsy (confirmation)
NS	Median 20.5 mo	NS • Albuminuria >3.5 g/24 h • Hypoalbuminemia • Hypercholesterolemia • Edema • Lipiduria	MN • Positive FAT1 (highly suggestive) Minimal change disease • History • Exclude infections, cancer, drugs Definitive • Kidney biopsy (confirmation)
CIN	After severe AKI (any cause); after chronic TA-TMA, BK virus nephropathy, obstruction and recurrent pyelonephritis	• Low-grade proteinuria • Progressive kidney failure	Presumptive • Clinical context Definitive • Kidney biopsy (confirmation)

References 6, 81, 123, 124, 126, 128–130. CIN, chronic interstitial nephritis; CNI, calcineurin inhibitor; FAT1, protocadherin FAT1; HSCT, hematopoietic stem cell transplant; LDH, lactate dehydrogenase; MN, membranous nephropathy; NS, nephrotic syndrome; TA-TMA, transplant-associated thrombotic microangiopathy.

Treatment of TA-TMA is discussed above and summarized in Table 6.

**Table 6:** Treatment approaches to the etiologies of CKD after hematopoietic stem cell transplantation

Etiology	Treatment
Chronic TA-TMA	Supportive measures 1. Treatment of hypertension (ACE or ARB) 2. Remove CNI if high suspicion of TMA induced by CNI • Be aware that this can result in exacerbation of GVHD • Substitute with MMF, steroids, and/or IL-2 receptor inhibitors 3. Avoid nephrotoxic agents (contrast, NSAIDs) 4. Dialysis if needed (refractory fluid overload, refractory hyperkalemia, metabolic acidosis, uremia)
CNI nephropathy	1. Reduce CNI exposure—suspend if possible 2. Substitute CNI for another drug (MMF, basiliximab, daclizumab)
NS	Supportive measures 1. Loop diuretics for fluid overload 2. ACEi or ARB for proteinuria 3. Anticoagulation as needed (according to KDIGO guidelines)
	Specific treatment 1. If GVHD+NS • Immunosuppression (CNI, rituximab, glucocorticoids, MMF) 2. MN • PLA2R antibody negative+FAT1 positive → rituximab • PLA2 antibody positive → rituximab (similar to patients with primary MN) • Glucocorticoids (mild response) 3. Minimal change disease • Oral glucocorticoids (as for primary minimal change disease, according to KDIGO guidelines) • Other suggestions: rituximab, CNI
CIN	Supportive measures 1. Treatment of hypertension (ACEi or ARB) 2. Avoid nephrotoxic agents (contrast, NSAIDs) 3. Dialysis if needed (refractory fluid overload, refractory hyperkalemia, metabolic acidosis, uremia)

Refs. 6,81,122,125,139. ACE, angiotensin-converting enzyme; ACEi, angiotensin-converting-enzyme inhibitor; ARB; angiotensin receptor blocker; CIN, chronic interstitial nephritis; CNI, calcineurin inhibitor; FAT1, protocadherin FAT1; GVHD, graft-versus-host-disease; HSCT, hematopoietic stem cell transplant; KDIGO, Kidney Disease Improving Global Outcomes; MMF, mycophenolate mofetil; MN, membranous nephropathy; NS, nephrotic syndrome; NSAIDs, nonsteroid anti-inflammatory drugs; PLA2R, phospholipase A2 receptor; TA-TMA, transplant-associated thrombotic microangiopathy.

#### CNI Toxicity

CNIs are usually administrated post-HSCT for short periods (3 months). However, in patients with GVHD, CNI exposure may be prolonged and lead to CKD.^6^ Mechanism of injury is similar to what occurs after solid organ transplantation, with vasoconstriction of afferent arterioles, endothelial vascular injury, tubular vacuolization, and interstitial fibrosis.^30^ Chronic hypoperfusion causes progressive/irreversible damage.

Patients typically present with progressive decline in kidney function, hypertension, and proteinuria (Table 5). Diagnosis is often presumptive in those with longstanding history of CNI administration. Kidney biopsy shows a pattern of glomerular FSGS and/or CIN with striped fibrosis.^123,124^

In patients with CKD due to CNI exposure, the drug should be discontinued if possible. Some authors recommend substituting mycophenolate mofetil, basiliximab, or even daclizumab to prevent aggravation of GVHD (Table 6).^6,125^

#### NS

Incidence of NS after HSCT is 1%–6%.^6,126,127^ Median onset of NS was 20.5 months (range, 3–174) in a series of 116 cases.^126^ Several works have revealed a close relationship between the development of NS shortly after cessation of immunosuppression and diagnosis of chronic GVHD. Most common histological findings are membranous nephropathy (65.5%), minimal change disease (19%), FSGS (7.7%), membranoproliferative GN (5.2%), and IgA nephropathy (2.6%).^126^

Recently, a novel protein, protocadherin FAT1, was identified as a target antigen in membranous nephropathy secondary to GVHD in the context of HSCT.^128,129^ Anti-phospholipase A2 receptor antibodies are typically negative.

Treatment of NS after HSCT is described in Table 6.^6,122^

#### CIN

As seen earlier, BK virus infection and TA-TMA may cause interstitial nephritis. Damage to the kidneys may persist leading to CIN (where fibrosis and tubular atrophy predominate).^130^ Severe AKI of any etiology may progress to irreversible tubulointerstitial fibrosis. Diagnosis and treatment of CIN are reviewed in Tables 5 and 6.^130^

### Special Considerations

#### HSCT in Patients with ESKD

Data regarding HSCT in patients with ESKD are limited and consist mostly of case reports and small case series. Martini *et al.* retrospectively studied seven hemodialysis patients who underwent AHSCT, showing 100% response rate and 0% treatment-related mortality at 12 months.^131^ In another study, eight patients requiring hemodialysis were submitted to NMA conditioning; patients who started hemodialysis right before HSCT had higher mortality rates, but patients in a regular hemodialysis program did not have adverse outcomes.^132^ Data regarding HSCT in patients on peritoneal dialysis are even more scarce. El Fakih *et al.* retrospectively studied of 24 patients (21 hemodialysis, three peritoneal dialysis) undergoing AHSCT for multiple myeloma; response rate was 92% with 0% treatment-related mortality at 1 year.^133^

#### HSCT after Kidney Transplantation

Patients with solid organ transplants have long-term exposure to immunosuppressants which increases risk for malignancies up to eight-fold in adults, with post-transplant lymphoproliferative disease having a reported incidence of 1.58 per 1000 person-years.^134^ Owing to long-term immunosuppressive therapy, there is also increased risk of relapse of hematological malignancy.^135^

HSCT after solid organ transplant presents several challenges: augmented risk of solid organ rejection after allogeneic HSCT (reaction of donor cells with the solid organ allograft); maintenance immunosuppression leading to greater risk of infection and malignancy relapse (due to poorer response to the HSCT); and uncertainty about the dosage for the conditioning regimen.

There are few reports of HSCT after kidney transplantation and they often have contradicting outcomes. A European study by Basak *et al.* included 28 patients with a solid organ transplant (12 with a kidney transplant) after allogeneic HSCT and demonstrated 40% 5-year survival with 33% solid organ failure at 12 months.^136^ A Japanese study published by Shinohara *et al.* included nine kidney transplant patients who underwent allogeneic HSCT: 8/9 progressed to severe allograft dysfunction with seven requiring maintenance hemodialysis; there were also high rates of infection.^135^

#### Kidney Transplantation after HSCT

HSCT may cause kidney damage with progression to CKD and ESKD, and kidney transplantation may be a viable option for selected patients. There are limited articles on this topic; these usually include patients with the same donor (both of hematopoietic stem cell and the kidney allograft) and have short follow-up periods. Brockmann *et al.* included 53 patients who had a kidney transplant after HSCT and observed 81% survival at a mean follow-up of 4 years and death of seven patients (two cardiac deaths, two malignancies, three infectious complications).^137^ Ziliotis *et al.* conducted a multicenter study which included 19 patients (with 16 receiving a kidney from a different donor than hematopoietic stem cell) and demonstrated 94% survival at 5 years; 18 patients had infectious complications; three patients developed malignancies.^138^ Simultaneous combined allogeneic BM and kidney transplant is described in, at least, one center and opens an exciting way of treating both malignancy and ESKD without ongoing immunosuppression.^114^ Therefore, kidney transplantation after HSCT appears to be relatively safe, with a good survival rate even though a higher risk of infections might be present.

## Conclusion

This work summarizes the broad spectrum of acute and chronic kidney complications of HSCT. Caring for these patients by a multidisciplinary team including onco-nephrology is essential.
